# Effects of mindful awareness intervention folk-dance intervention and combined intervention on academic burnout in left-behind children in rural China

**DOI:** 10.3389/fpsyg.2026.1745058

**Published:** 2026-04-27

**Authors:** Huiting Ren, Qian Yang, Jiaxi Chen, Yiping Luo, Xiao Liu, Chunxia Lu, Qiang Liu

**Affiliations:** 1 School of Music and Dance, Hunan City University, Yiyang, China; 2 Department of Sport Education, Hunan Normal University, Changsha, China; 3 Department of Physical Education, Central South University, Changsha, China

**Keywords:** academic burnout, combined intervention, folk-dance intervention, left-behind children, mindful awareness intervention

## Abstract

**Objectives:**

This study examined the comparative effectiveness of three distinct interventions—a folk-dance intervention, a mindful awareness intervention, and a combined intervention integrating both strategies—in alleviating academic burnout among a sample of rural Chinese left-behind children.

**Methods:**

A total of 100 rural left-behind children (54 boys, 46 girls; age = 10.4 ± 0.9) were randomly assigned to four groups: mindful awareness training, folk-dance intervention, combined intervention, and control. Each group received four sessions per week for 12 weeks. Academic burnout was assessed at three time points: baseline (T_0_), post-intervention (T_1_) and 6-week follow-up (T_2_). Data were analyzed using repeated-measures ANOVA with Bonferroni correction.

**Results:**

Significant group differences were observed in total academic burnout. A repeated-measures ANOVA revealed a significant Group × Time interaction, *F* (6, 192) = 43.89, *p* < 0.001, ηp^2^ = 0.57, indicating that changes in academic burnout differed across groups over time. A significant main effect of Time was also found, *F* (2, 192) = 303.44, *p* < 0.001, ηp^2^ = 0.76. Across the intervention period, all treatment groups showed significant reductions in burnout scores compared with the control group. Among the interventions, the combined dance-mindful awareness group demonstrated the largest and most sustained reduction in burnout, showing the lowest burnout levels at follow-up. Similar patterns were observed for the subdimensions of burnout, including physical exhaustion, emotional exhaustion, learning attitude, and reduced learning efficacy (all *p* < 0.001).

**Conclusion:**

These findings suggest that the integration of folk-dance and mindful awareness produces stronger and more sustained reductions in academic burnout than either approach alone, providing a culturally sensitive and evidence-based intervention strategy for addressing academic burnout among left-behind children.

## Introduction

Over the past few decades, China’s rapid urbanization and industrialization have led to substantial internal migration, as millions of rural laborers relocate to cities in search of better employment opportunities (Zhaojun and Nengsheng, 2008). Due to the high cost of urban living and limited earning capacity, many migrant parents are unable to bring their children with them (Zhang and Wu, 2017). As a result, a large number of children remain in rural areas under the care of extended family members, predominantly grandparents (Chan, 2010). According to the State Council of China, left-behind children refer to rural minors under the age of 16 who have both parents—or one migrant parent and another incapable guardian—absent from home due to migration (Wen et al., 2021). Despite years of policy attention, the number of such children remains high, with approximately 9.02 million still living apart from both parents as of 2023 (Ministry of Civil Affairs of the People’s Republic of China, 2023).

Prolonged parental absence can undermine not only emotional wellbeing but also academic performance. Without consistent parental support in educational activities, such as homework supervision and school engagement, these children face elevated learning challenges (Zhu et al., 2023). Moreover, emotional detachment caused by familial separation often diminishes students’ intrinsic motivation, which may heighten the risk of academic burnout. Academic burnout is broadly conceptualized as a maladaptive response to educational stress, marked by emotional exhaustion, academic disengagement, and feelings of ineffectiveness (Lian, 2005; Schaufeli et al., 2002). Its consequences extend beyond poor academic outcomes, often manifesting in mental health deterioration, psychosomatic symptoms, and increased vulnerability to depression (Lee and Lee, 2018; May et al., 2015). Left-behind children, lacking emotional security and parental guidance, are particularly susceptible to these outcomes. Recent empirical data underscore this concern: a nationwide study by Zhou et al. (2020) revealed that nearly 38% of left-behind students in rural China suffer from moderate to severe levels of academic burnout—significantly higher than their peers living with both parents. This disparity emphasizes the pressing need for targeted, evidence-based interventions.

Mindful awareness, rooted in Eastern contemplative traditions, has evolved into a structured psychological intervention that emphasizes present-moment awareness and non-reactive attention (Prazak et al., 2012). Previous research suggests that mindful awareness practices may contribute to improvements in emotional regulation and stress reduction, which are conceptualized in the present study as theoretical mechanisms underlying intervention effects rather than directly measured outcomes. In school settings, mindful awareness interventions (MBIs) have demonstrated effectiveness in mitigating academic burnout by enhancing cognitive control and emotional balance (Wang et al., 2024). However, traditional MBIs often involve static meditation practices, which may lack immediate feedback and engagement—posing challenges for populations such as left-behind children, who are already prone to attentional fatigue and disengagement.

In contrast, folk-dance interventions incorporate rhythmic movement, music, and group interaction, offering a dynamic, culturally relevant alternative. As a form of aerobic exercise, folk-dance has been associated with reductions in stress-related biomarkers, such as cortisol, and improved emotional states via endorphin release (Koch et al., 2014). Participation in structured dance programs has also been linked to enhanced coping strategies and psychological wellbeing among adolescents (Joseph et al., 2014). Moreover, recent evidence further supports the mental health benefits of dance-based interventions. For example, Norouzi et al. (2020) demonstrated that Zumba dancing and aerobic exercise significantly improved working memory, motor function, and depressive symptoms in women with fibromyalgia, highlighting the broader therapeutic potential of rhythmic movement in enhancing both physical and emotional wellbeing. This addition extends the existing evidence base by underscoring that dance interventions—whether in clinical or community contexts—can yield meaningful psychological and cognitive benefits, providing further justification for their integration in youth mental health programs. Importantly, in rural Chinese contexts, folk dance is often a familiar and socially valued activity. The decision to focus on mindful awareness and folk-dance stems from both theoretical grounding and contextual appropriateness. Mindful awareness helps cultivate emotional stability and attentional regulation, addressing internalized stressors. Meanwhile, folk dance engages students physically and socially, promoting belonging and embodied resilience. For left-behind children—who often experience emotional neglect and social isolation—this dual-focus approach targets both internal and external dimensions of academic burnout (Huang and Noknoi, 2025).

While both mindfulness awareness and folk-dance interventions show independent benefits, concerns remain regarding the long-term sustainability of single-modality approaches. For example, Zhang et al. (2024) reported that although folk-dance participation significantly reduced academic burnout during implementation, these effects diminished within 3 months post-intervention. Such findings raise questions about whether unimodal interventions provide durable protection against burnout in high-risk youth. Consequently, increasing attention has been directed toward multimodal interventions that integrate cognitive and physical components. Prior studies indicate that combining mindful awareness with physical activity yields greater improvements in emotional regulation, executive function, and stress tolerance than either approach alone (De Bruin et al., 2017; Del Giorno et al., 2010). In the present study, these constructs are discussed as potential explanatory mechanisms, whereas academic burnout served as the primary outcome variable. Additionally, emerging school-based research suggests that mindfulness can be effectively integrated with other modalities—such as movement-based or arts-based activities—and may produce meaningful improvements in student engagement, emotional regulation, and wellbeing (Coholic et al., 2020; U et al., 2025; Wong et al., 2024). These constructs are referenced here to provide theoretical context rather than as directly measured outcomes in the present study. These findings provide further support for adopting multimodal intervention frameworks in educational contexts. Theoretically, mindful awareness and folk-dance operate through complementary regulatory mechanisms. Mindful awareness primarily engages top-down processes by strengthening executive control, attentional stability, metacognitive awareness, and present-moment monitoring, thereby reducing rumination and enhancing psychological wellbeing (Lutz et al., 2008; Shapiro et al., 2006). In contrast, folk dance activates bottom-up pathways through embodied movement, rhythmic synchronization, music, and group interaction, which promote physiological activation, positive affect, and social connectedness (Coholic et al., 2020; Koch et al., 2019; Murrock and Higgins, 2009). When integrated, these modalities are not merely additive; rather, mindful awareness deepens bodily awareness during movement, while dance sustains engagement and emotional vitality through physical expression, grounding abstract attentional skills in concrete sensory experience (Basso and Suzuki, 2016). This reciprocal interaction forms a unified mind-body regulatory system that simultaneously enhances cognitive control and embodied emotional engagement. Such integration may be particularly relevant for left-behind children, who often experience both cognitive strain and emotional deprivation. Addressing only one regulatory pathway may therefore be insufficient. From a developmental perspective, late childhood (9–11 years) is characterized by rapid maturation of executive functions alongside heightened sensitivity to peer interaction and belonging. Integrating mindful awareness with socially synchronized movement may thus align closely with the neurocognitive and socio-emotional characteristics of this age group, providing a coherent theoretical foundation for examining the combined intervention as a potentially more durable strategy for reducing academic burnout.

Accordingly, the present study aims to evaluate the effectiveness of folk-dance and mindful awareness interventions—both individually and in combination—in reducing academic burnout among left-behind children in rural China. Academic burnout was specified as the primary and sole outcome variable in this study. Drawing on a mind-body integrative framework, constructs such as emotional regulation and self-efficacy are conceptualized as potential theoretical mechanisms through which these interventions may exert their effects, rather than as directly measured outcomes. By comparing the short-term and sustained impacts of these interventions, this research seeks to identify the most effective and culturally responsive strategies to support the educational and psychological wellbeing of this vulnerable population.

## Materials and methods

### Participants

**FIGURE 1:**
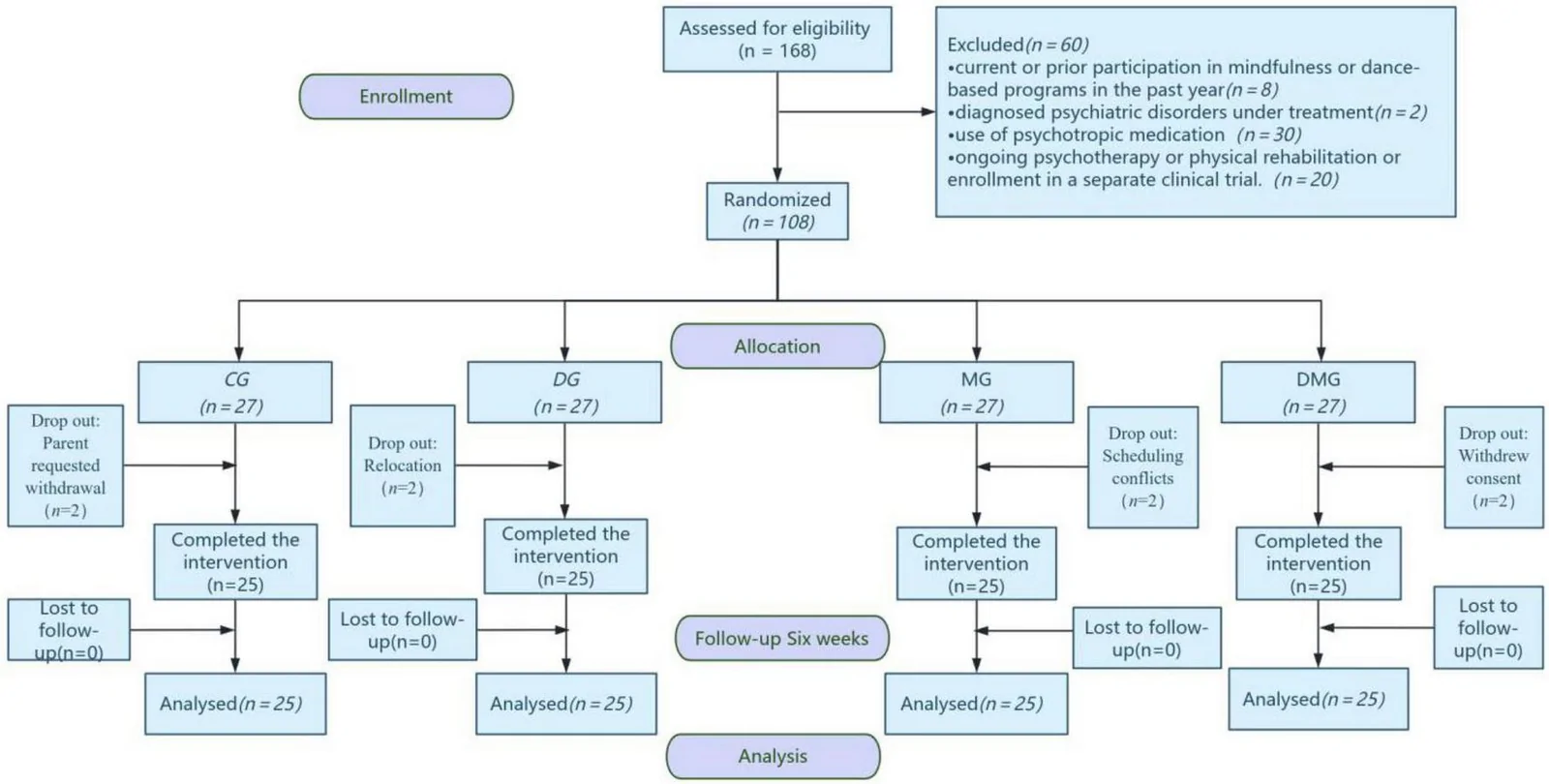
Flow diagram of participant recruitment, randomization, and analysis.

Prior to data collection, a power analysis was conducted using G*Power software (version 3.1.9.7) to determine the required sample size. Assuming a statistical power of 0.90, an alpha level of 0.05, and an expected effect size of *f* = 0.32 derived from a previous school-based mindful awareness and dance intervention study (Lau and Hue, 2011), the minimum required sample size was estimated to be 84 participants (21 per group). To account for potential attrition, 108 eligible students were randomized into four groups (*n* = 27 per group). Participants were sorted alphabetically by surname and randomly assigned using a computer-generated randomization sequence created through Randomizer.org. The allocation sequence was generated by a researcher who was not involved in recruitment, assessment, or intervention delivery. Group assignments were placed in sequentially numbered, opaque, sealed envelopes to ensure allocation concealment. During the intervention period, eight participants withdrew from the study after randomization due to parent-requested withdrawal (*n* = 2), relocation (*n* = 2), scheduling conflicts (*n* = 2), and withdrawal of consent (*n* = 2). Consequently, 100 participants completed the intervention and follow-up assessments and were included in the final analysis (*n* = 25 per group). The participant recruitment, allocation, and attrition process is presented in the CONSORT flow diagram (Figure 1). Baseline questionnaire data were available for the participants who completed the intervention and were included in the final analysis.

### Recruitment and setting

Participants were recruited from Grades 4 and 5 at Huangdu Primary School, Shaodong City, Hunan Province, China. Recruitment was conducted between March 2023 and April 2023, prior to the initiation of the intervention program. This age range (9–11 years) was selected because late childhood represents a critical developmental stage for emotional regulation, attention, and social interaction (Blakemore and Choudhury, 2006). Children at this stage possess sufficient cognitive maturity to engage in mindful awareness practice while remaining receptive to embodied and play-based activities such as folk-dance. Recruitment was conducted in collaboration with school administrators and classroom teachers, who distributed study information sheets and consent forms to guardians of eligible left-behind students. Participation was voluntary. Guardians and children were explicitly informed, both verbally and in writing, that participation was entirely voluntary and that participants could withdraw from the study at any time without academic penalty, loss of benefits, or any negative consequences. Only students who met the inclusion criteria and returned written parental consent and child assent were enrolled in the study.

### Eligibility criteria

Eligibility criteria included: (I) a left-behind child designation, defined according to China’s Ministry of Civil Affairs as a rural child under 16 with both parents, or one parent and another incapable guardian, living and working away from home for 6 months or longer. This “under 16” definition reflects the national administrative policy classification of left-behind children; however, the present study specifically restricted recruitment to students enrolled in Grades 4 and 5, whose actual ages ranged from 9 to 11 years. No children aged 12–16 years were included in the final sample; (II) current enrollment in Grades 4 or 5 at the recruitment site; and (III) scoring above the clinical threshold on the Learning Burnout Scale (total score ≥ 43), consistent with prior research using this instrument. Only children formally identified as left-behind were eligible to participate. This status was verified through school records and confirmed by guardians, who provided supporting documentation of parental migration or employment outside the local area. Non-left-behind children attending the same school were not included in the recruitment process.

Exclusion criteria included: (I) current or prior participation in mindful awareness or dance-based programs in the past year; (II) diagnosed psychiatric disorders under treatment (e.g., major depression, ADHD); (III) use of psychotropic medication; (IV) ongoing psychotherapy or physical rehabilitation or enrollment in a separate clinical trial; (V) presence of medical conditions that contraindicate moderate physical activity or restrict safe participation in dance-based exercise (e.g., severe cardiovascular disease, musculoskeletal disorders).

### Study design

This study employed a parallel-group, evaluator-blinded randomized controlled trial (RCT) design. The intervention lasted 12 weeks and consisted of four 40-min sessions per week (Monday–Thursday, 16:00–16:40), totaling approximately 32 h of guided training. Participants in the three intervention arms attended structured, instructor-led sessions in designated classrooms. The control group maintained their usual extracurricular routine. The study was approved by the Ethics Committee of Hunan Normal University, School of Life and Pharmaceutical Sciences (Approval No. 2023-008; February 23, 2023). All procedures adhered to the Declaration of Helsinki. Written informed consent was obtained from guardians, and child assent was obtained using age-appropriate explanations. The trial was retrospectively registered in the Chinese Clinical Trial Registry (ChiCTR2500098927; March 17, 2025).

### Trial registration and study timeline

The study protocol was approved by the Ethics Committee of Hunan Normal University on February 23, 2023. Participant recruitment was conducted between March and April 2023. The 12-week intervention program was implemented from April 2023 to July 2023. Post-intervention assessments (T_1_) were completed immediately after the intervention period, and follow-up assessments (T_2_) were conducted 6 weeks later in August 2023. Data cleaning and database locking were completed in September 2023, after which statistical analyses were initiated. The trial was retrospectively registered in the Chinese Clinical Trial Registry (ChiCTR2500098927) on March 17, 2025. The registered protocol specified academic burnout as the primary outcome, assessed at baseline (T_0_), post-intervention (T_1_), and follow-up (T_2_). The planned analyses included repeated-measures comparisons across groups and time points. These registered outcomes, time points, and analytical approaches are consistent with those reported in the present manuscript.

### Intervention fidelity and safety monitoring

The intervention protocol was developed by an interdisciplinary team consisting of a professor of sport psychology and rehabilitation and eight graduate students (four specializing in psychology and four in dance education). The mindful awareness sessions were delivered by graduate students in psychology under the supervision of the senior professor specializing in sport psychology and rehabilitation. Although the instructors did not hold formal certification in standardized mindfulness-based programs (e.g., MBSR), they received systematic training in mindfulness theory and practice as part of their graduate coursework and research preparation. Prior to the intervention, all mindfulness facilitators completed structured training specific to this study, which included: (a) intensive workshops on the theoretical foundations of mindful awareness, (b) guided practice sessions to standardize instructional language and pacing, (c) rehearsal of the full intervention protocol using the study manual, and (d) supervised mock sessions with feedback provided by the supervising professor. The folk-dance sessions were delivered by graduate students specializing in dance education. These instructors had formal academic training in dance pedagogy and prior experience conducting structured dance activities in school settings. To ensure intervention fidelity, all facilitators underwent standardized preparation prior to program implementation. A detailed intervention manual was developed outlining session structure, duration, instructional content, and activity progression. Session adherence was monitored using structured checklists completed after each session, and periodic supervision meetings were held to maintain consistency across groups. Throughout the intervention period, the supervising professor conducted regular supervision meetings to review session implementation, address challenges, and ensure adherence to the protocol. Participant safety was closely monitored. Teachers and school health staff documented any signs of discomfort, injury, or distress in an incident log. No adverse events were reported during the intervention or follow-up periods.

## Interventions

All intervention programs were conducted over a 12-week period, with four sessions per week, and each session lasting 40 min (16:00–16:40). The structure and time allocation of each intervention session were standardized to ensure comparable exposure across groups. A summary of the intervention structure is presented in Table 1.

**TABLE 1:** Structure and time allocation of intervention sessions across groups.

Group	Session components	Time (minutes)	Description
DG	Warm-up	5	Gentle joint mobilization and low-intensity movements to prepare for physical activity
	Folk-dance practice	30	Rhythmic movement, including circle formations, partner coordination, and group choreography
	Cool-down	5	Stretching and flexibility exercises for relaxation and recovery
	Total	40	
MG	Guided introduction	5	Session theme explanation and attentional settling
	Mindfulness practice	30	Mindful breathing, body scan, five-senses grounding, emotion awareness, loving-kindness reflection
	Reflection/sharing	5	Brief guided reflection and voluntary sharing of experiences
	Total	40	
DMG	Mindful awareness practice (including brief reflection)	20	Condensed mindfulness practice including breath awareness, body scan, and embedded reflection (2–3 min)
	Folk-dance practice	20	Folk-dance activities incorporating attention-guiding cues and embodied awareness
	Total	40	
CG	Regular school activities	–	No intervention provided during the study period

### Folk-dance group

**FIGURE 2:**
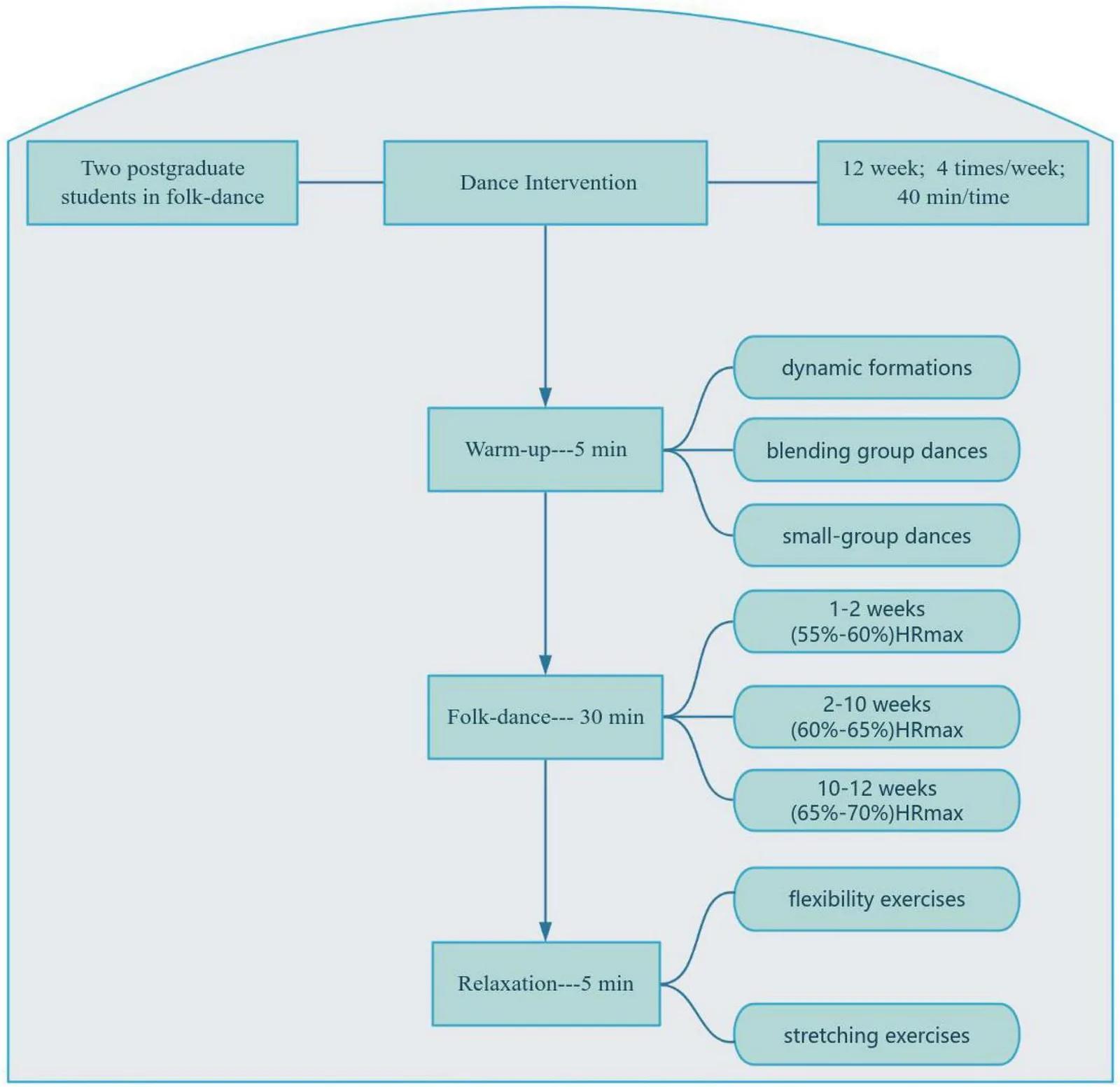
Flowchart of working folk-dance intervention.

Participants in the folk-dance group received a structured folk-dance intervention designed to promote physical activity, emotional expression, and social interaction. Each 40-min session consisted of three phases. First, a 5-min warm-up was conducted, including gentle joint mobilization and low-intensity movements to prepare participants for physical activity and reduce injury risk. Second, participants engaged in approximately 30 min of folk-dance practice. The dance sessions incorporated dynamic formations, including circle dances, parallel lines, partner coordination, and small-group choreography. The intensity of the movements gradually increased over the course of the program. Emphasis was placed on rhythm, spatial awareness, and cooperative movement. Variations such as directional changes, faster turns, and modified hand positions were introduced to maintain engagement. During dance practice, strict silence was not required. Brief verbal coordination and instructional cues were permitted to facilitate synchronization and group interaction. When movement errors occurred, instructors provided supportive feedback and encouraged gradual adjustment rather than corrective criticism, thereby fostering a psychologically safe learning environment. Finally, each session concluded with a 5-min cool-down period, consisting of stretching and flexibility exercises to promote relaxation and recovery. To monitor exercise intensity, participants wore portable heart-rate monitors and maintained moderate intensity levels (55–70% HRmax). Small incentives were periodically provided to encourage sustained participation (see Figure 2).

### Mindful awareness group

**FIGURE 3:**
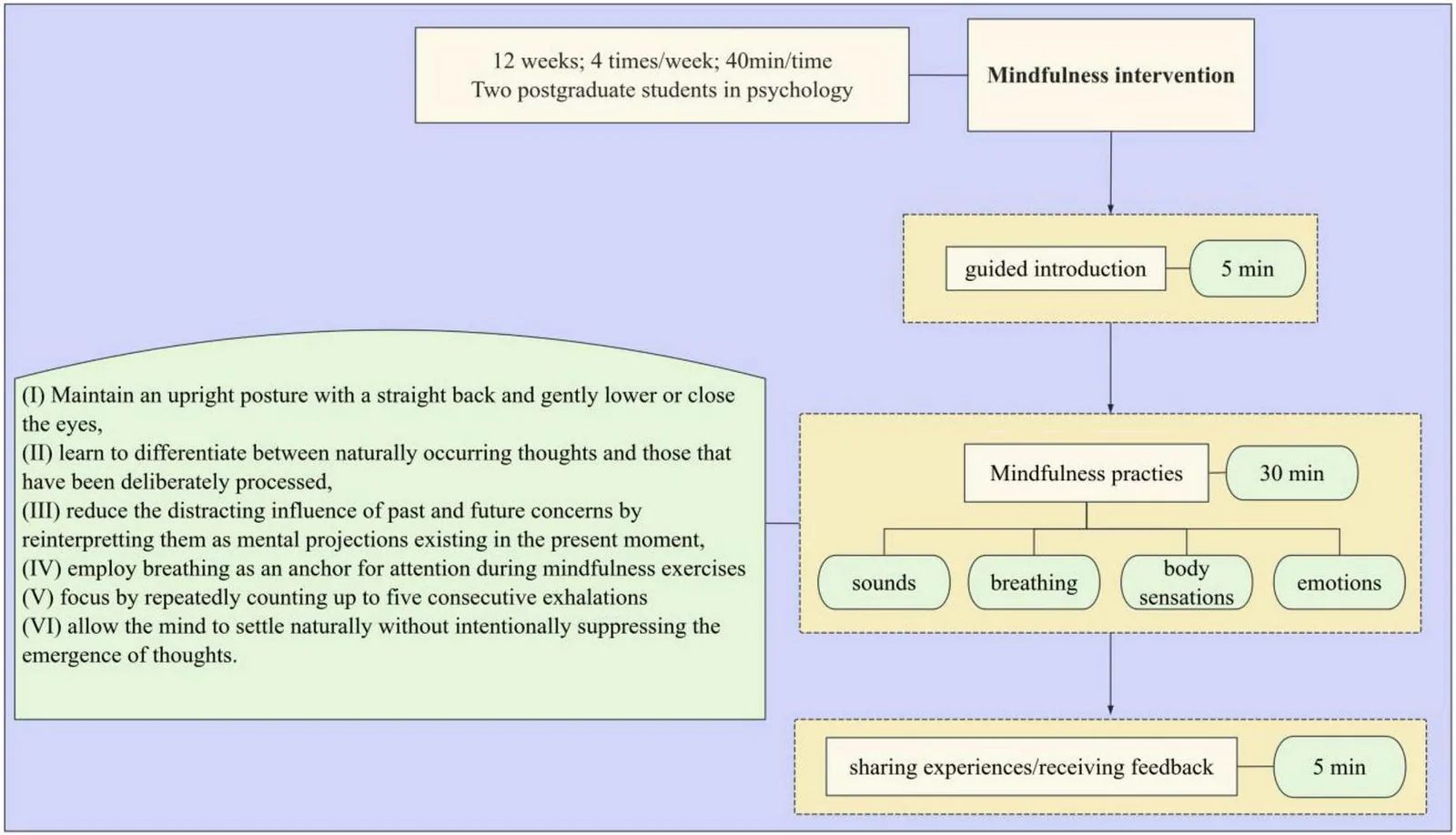
Flowchart of working with mindful awareness intervention.

Participants in the mindful awareness group followed a school-based mindfulness curriculum adapted from Calmer Choice and Mind UP (Schonert-Reichl and Lawlor, 2010), with modifications to ensure developmental appropriateness for children. Each 40-min session consisted of three structured components. First, a 5-min guided introduction was provided, during which instructors introduced the theme of the session and guided participants in settling their attention using simple and age-appropriate explanations. Second, participants engaged in approximately 30 min of structured mindfulness practice. Core exercises included mindful breathing, brief body scan exercises, five-senses grounding activities, emotion labeling, and loving-kindness reflections. Age-appropriate metaphors (e.g., “breathing with a balloon”) and sensory-based activities were used to enhance comprehension and engagement. Silence was generally encouraged during breathing and body scan exercises to support attentional focus. When distractions occurred, instructors gently redirected attention using non-judgmental language (e.g., “Notice where your mind went and bring it back to your breath”). Finally, each session concluded with approximately 5 min of guided reflection, during which participants were invited to voluntarily share brief observations about their breathing, bodily sensations, or emotional experiences. Instructors provided supportive feedback and brief summaries to reinforce mindful awareness skills (see Figure 3).

### Combined group

Participants in the combined group received an integrated intervention combining mindful awareness and folk-dance practice. Consistent with the overall study design, each session lasted 40 min. Each session included 20 min of mindful awareness practice followed by 20 min of folk-dance training, ensuring that the total duration and intervention exposure were equivalent to the other intervention groups. The mindful awareness component followed the same core practices used in the MG group (breath awareness, body scan, emotion awareness, and loving-kindness reflection), delivered in a condensed format. Within this 20-min mindfulness segment, approximately 2–3 min of brief structured reflection were incorporated as part of the mindfulness practice, during which participants were invited, on a voluntary basis, to share short observations about their breathing, bodily sensations, or emotional experiences. Facilitators used simple guiding prompts (e.g., “What did you notice about your breathing?,” “Did anything feel easier or more difficult today?,” or “What did you enjoy about today’s activity?”). This reflection period was embedded within the mindfulness segment and did not extend the overall session duration. The second half of the session consisted of 20 min of folk-dance practice, following the same instructional structure used in the DG group. Although the intervention followed a sequential structure, mindful awareness principles were intentionally incorporated into the dance component. Instructors used attention-guiding cues such as “notice your breathing as you move” and “feel your feet contacting the ground” to encourage embodied awareness during movement. When coordination difficulties occurred, participants were encouraged to observe the experience without self-criticism and gradually adjust their movements, consistent with the non-judgmental stance cultivated during mindfulness practice. To ensure intervention fidelity, facilitators followed a standardized intervention outline and session checklist throughout the program.

### Control group

The control group was randomly allocated at baseline and did not receive any specific intervention. However, they were subjected to the same monitoring procedures as the experimental group to ensure consistency in data collection and observation.

### Measurement

Academic burnout was assessed using the Learning Burnout Scale (LBS) developed by Wu et al. (2010), which has been widely used in educational and psychological research in China. The scale consists of 21 items rated on a 5-point Likert scale (1 = strongly disagree to 5 = strongly agree). In the present study, the scale was organized into four subdimensions: Physical Exhaustion (PE), Emotional Exhaustion (EE), Attitudes toward Learning (ATT), and Reduced Learning Efficacy (RLE). Scores for each dimension were calculated by summing the respective items, and the total score was derived from the sum of all items, with higher scores indicating higher levels of academic burnout. Participant eligibility for the study was determined separately using the predefined inclusion criterion of a total burnout score ≥ 43, as described in the Eligibility Criteria section. The scale includes several reverse-scored items (Items 5, 7, 8, 10, 11, and 12), which were recoded prior to analysis. Previous validation studies have confirmed the robust psychometric properties of the LBS among Chinese student populations, including good construct validity (supported by confirmatory factor analysis with excellent model fit indices: χ^2^/df = 4.044, RMSEA = 0.04, CFI = 0.958, TLI = 0.949) and discriminant validity (Tang et al., 2021). In the present study, the scale demonstrated excellent internal consistency. The overall Cronbach’s α was 0.895, and the Cronbach’s α values for the four subscales were as follows: PE (0.809), EE (0.871), ATT (0.878), and RLE (0.820). For the detailed validation process and psychometric properties of the Learning Burnout Scale, please refer to Supplementary material.

### Statistical analysis

Statistical analyses were conducted using IBM SPSS Statistics 26.0. Figures were generated using GraphPad Prism 9. Analyses followed a per-protocol approach, including only participants who completed the intervention and follow-up assessments (*n* = 100). No re-randomization occurred after withdrawals, and missing data were not imputed. Continuous variables are presented as mean ± SD and categorical variables as counts and percentages. Baseline differences across groups were assessed using one-way ANOVA for continuous variables and chi-square tests for categorical variables. A 4 (Group: CG, DG, MG, DMG) × 3 (Time: T_0_, T_1_, T_2_) repeated-measures ANOVA was used to evaluate intervention effects over time (pre-intervention, post-intervention, and 6-week follow-up). *Post-hoc* pairwise comparisons were adjusted using Bonferroni correction. To convey practical as well as statistical significance, effect sizes are reported alongside *p*-values for all primary analyses. For ANOVA effects, partial eta squared (ηp^2^) was reported. For pairwise comparisons, Cohen’s *d* was calculated as the standardized mean difference between groups using pooled standard deviations. Effect-size interpretation followed conventional benchmarks (Cohen, 1988): Cohen’s *d* = 0.20 (small), 0.50 (medium), and 0.80 (large). For partial eta squared (ηp^2^), effect sizes were interpreted as small (0.01), medium (0.06), and large (0.14).

## Results

### Baseline characteristics of participants

Of the 108 randomized participants, eight withdrew during the intervention period. Consequently, 100 participants completed the intervention and were included in the final analysis (*n* = 25 per group). Baseline sociodemographic and psychological characteristics of participants are presented in Table 2. The assessed variables included gender, age, parental marital status, only child status, household income, and baseline academic burnout scores. Across all measured variables, no statistically significant differences were observed among the four groups at baseline (all *p* > 0.05), indicating successful randomization and comparability between groups prior to the intervention. Specifically, the distribution of gender, age, family structure, and socioeconomic indicators was comparable across the control (CG), dance (DG), mindful awareness (MG), and combined intervention (DMG) groups. Baseline academic burnout and its subdimensions (PE, EE, ATT, RLE), assessed prior to the intervention, also did not differ significantly between groups, further supporting initial equivalence.

**TABLE 2:** Baseline characteristics of participants across the four groups (*n* = 100) [*M* ± SD, *n* (%)].

Variable	Characteristic	CG (*n* = 25)	DG (*n* = 25)	MG (*n* = 25)	DMG (*n* = 25)	F/χ ^2^	*p*
Gender	Male	14 (56.0%)	13 (52.0%)	12 (48.0%)	14 (56.0%)	0.16	0.98
	Female	11 (44.0%)	12 (48.0%)	13 (52.0%)	11 (44.0%)		
Age	Years	10.52 ± 0.77	10.24 ± 0.87	10.20 ± 0.86	10.56 ± 0.96	1.14	0.33
Only child	Yes	16 (64.0%)	15 (60.0%)	17 (68.0%)	16 (64.0%)	0.34	0.95
	No	9 (36.0%)	10 (40.0%)	8 (32.0%)	9 (36.0%)		
Parental marital status	Married	15 (60.0%)	13 (52.0%)	14 (56.0%)	12 (48.0%)	2.52	0.98
	Divorced	6 (24.0%)	6 (24.0%)	5 (20.0%)	5 (20.0%)		
	Remarried	2 (8.0%)	3 (12.0%)	1 (4.0%)	2 (12.0%)		
	Widowed	2 (8.0%)	3 (12.0%)	1 (4.0%)	2 (12.0%)		
Household income (Yuan/year)	< 5,000	4 (16.0%)	3 (12.0%)	4 (16.0%)	4 (16.0%)	1.76	0.99
	< 30,000	7 (28.0%)	8 (32.0%)	7 (28.0%)	7 (28.0%)		
	< 80,000	11(44.0%)	11 (44.0%)	13 (52.0%)	12 (48.0%)		
	> 80,000	3 (12.0%)	3 (12.0%)	1 (4.0%)	2 (8.0%)		
Academic burnout	Total	53.00 ± 18.33	49.12 ± 14.77	55.64 ± 17.11	52.48 ± 15.88	0.65	0.58
	PE	10.56 ± 3.86	10.56 ± 3.31	11.32 ± 4.48	10.84 ± 3.76	0.21	0.88
	EE	19.60 ± 7.52	17.52 ± 5.79	19.72 ± 8.41	19.64 ± 7.16	0.53	0.65
	ATT	9.28 ± 3.73	8.32 ± 3.33	10.48 ± 4.77	9.24 ± 4.25	1.18	0.31
	RLE	13.56 ± 4.89	12.72 ± 4.14	14.12 ± 3.67	12.76 ± 4.07	0.63	0.59

CG, Control group; DG, Dance group; MG, mindful awareness group; DMG, Combined intervention group.

### Effects of interventions on academic burnout

**FIGURE 4:**
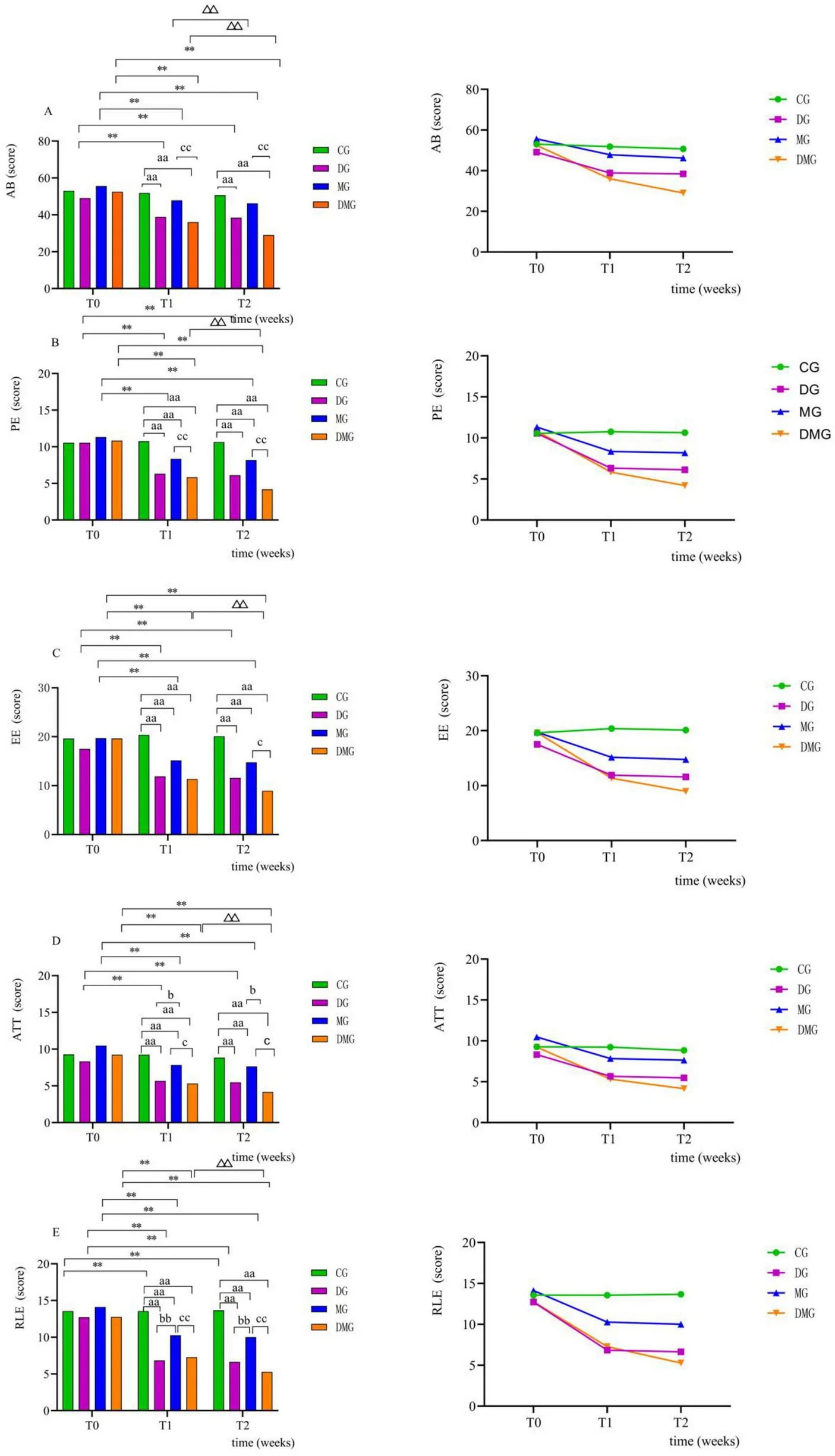
Effects of different interventions on academic burnout. **(A)** AB represents total academic burnout. **(B)** PE represents physical exhaustion. **(C)** EE represents emotional exhaustion. **(D)** ATT represents attitudes toward learning. **(E)** RLE represents reduced learning efficacy. **Indicates *p* < 0.001 compared with T0; △△ indicates *p* < 0.001 compared with T1; “a–c” indicate between-group comparisons, where: “a” indicates comparison with the control group (CG), “b” indicates comparison with the dance group (DG), and “c” indicates comparison with the mindful awareness group (MG).

Table 3 presents the means and standard deviations of total academic burnout (AB) and its four subdimensions—physical exhaustion (PE), emotional exhaustion (EE), attitude toward learning (ATT), and reduced learning efficacy (RLE)—across four groups at baseline (T_0_), after the intervention (T_1_), and at follow-up (T_2_). A mixed-design analysis of variance revealed significant Group × Time interactions for total AB, *F* (6, 192) = 43.89, *p* < 0.001, ηp^2^ = 0.57, indicating that the magnitude of change over time differed across groups. Significant interaction effects were also found for PE, *F* (6, 192) = 37.57, *p* < 0.001, ηp^2^ = 0.54; EE, *F* (6, 192) = 34.45, *p* < 0.001, ηp^2^ = 0.51; ATT, *F* (6, 192) = 15.79, *p* < 0.001, ηp^2^ = 0.33; and RLE, *F* (6, 192) = 27.38, *p* < 0.001, ηp^2^ = 0.46. Accordingly, follow-up simple effects analyses were conducted. As shown in Figure 4, burnout scores generally declined over time in the intervention groups, although the trajectories differed across interventions.

**TABLE 3:** Effects of different interventions on academic burnout.

Variable	Group	T_0_	T_1_	T_2_	F/η ^2^/*p* group	F/η ^2^/*p* time	F/η ^2^/*p* group × time
LBS total	CG	53.00 ± 18.33	51.80 ± 17.42	50.68 ± 16.37	4.77/0.13/ <0.001**	303.44/0.76/ <0.001**	43.89/0.57/ <0.001**
	DG	49.12 ± 14.77	38.92 ± 10.85^aa^**	38.40 ± 10.81^aa^**			
	MG	55.64 ± 17.11	47.80 ± 13.76**	46.24 ± 12.77**^△△^			
	DMG	52.48 ± 15.88	36.04 ± 9.71^aacc^**	29.04 ± 7.06^aacc^**^△△^			
PE	CG	10.56 ± 3.86	10.76 ± 3.75	10.64 ± 3.77	7.75/0.19/ <0.001**	238.35/0.71/ <0.001**	37.57/0.54/ <0.001**
	DG	10.56 ± 3.31	6.32 ± 1.88^aa^**	6.12 ± 1.85^aa^**			
	MG	11.32 ± 4.48	8.36 ± 3.08^aa^**	8.20 ± 3.14^aa^**			
	DMG	10.84 ± 3.76	5.84 ± 1.84^aacc^**	4.20 ± 0.50^aacc^**^△△^			
EE	CG	19.60 ± 7.52	20.40 ± 7.90	20.12 ± 7.28	7.37/0.18/ <0.001**	179.66/0.65/ <0.001**	34.45/0.51/ <0.001**
	DG	17.52 ± 5.79	11.92 ± 2.81^aa^**	11.60 ± 2.62^aa^**			
	MG	19.72 ± 8.41	15.16 ± 6.47^aa^**	14.76 ± 5.96^aa^**			
	DMG	19.64 ± 7.16	11.36 ± 2.95^aa^**	8.96 ± 1.45^aac^**^△△^			
ATT	CG	9.28 ± 3.73	9.24 ± 3.44	8.84 ± 3.35	5.98/0.15/ <0.001**	131.37/0.57/ <0.001**	15.79/0.33/ <0.001**
	DG	8.32 ± 3.33	5.68 ± 1.81^aa^**	5.48 ± 1.61^aa^**			
	MG	10.48 ± 4.77	7.84 ± 3.42^b^**	7.64 ± 3.42^b^**			
	DMG	9.24 ± 4.25	5.32 ± 1.84^aac^**	4.16 ± 0.47^aac^**^△△^			
RLE	CG	13.56 ± 4.89	13.56 ± 4.82	13.68 ± 5.28	14.81/0.31/ <0.001**	195.88/0.67/ <0.001**	27.38/0.46/ <0.001**
	DG	12.72 ± 4.14	6.84 ± 1.67^aa^**	6.64 ± 1.60^aa^**			
	MG	14.12 ± 3.67	10.28 ± 2.50^aabb^**	10.00 ± 2.34^aabb^**			
	DMG	12.76 ± 4.07	7.28 ± 1.96^aacc^**	5.28 ± 0.67^aacc^**^△△^			

a < 0.05, aa < 0.001, compared with CG; b < 0.05, bb < 0.001, compared with DG; c < 0.05, cc < 0.001 compared with MG;

*p < 0.05,

**p < 0.001, compared with T_0_; △*p* < 0.05,

△△*p* < 0.001, compared with T_1_.

Significant main effects of Time were observed for total AB, *F* (2, 192) = 303.44, *p* < 0.001, ηp^2^ = 0.76, as well as for PE, *F* (2, 192) = 238.35, *p* < 0.001, ηp^2^ = 0.71; EE, *F* (2, 192) = 179.66, *p* < 0.001, ηp^2^ = 0.65; ATT, *F* (2, 192) = 131.37, *p* < 0.001, ηp^2^ = 0.57; and RLE, *F* (2, 192) = 195.88, *p* < 0.001, ηp^2^ = 0.67. Simple effects analyses indicated that the control group (CG) showed no significant changes from T_0_ to T_1_ or T_2_ across AB and all subdimensions (all *p* > 0.05), suggesting stable burnout levels across the assessment period. In contrast, all intervention groups (DG, MG, and DMG) showed significantly lower AB, PE, EE, ATT, and RLE scores at both T_1_ and T_2_ compared with T_0_ (all *p* < 0.05), indicating sustained improvements following the interventions.

Significant main effects of Group were also observed for total AB, *F* (3, 96) = 4.77, *p* < 0.001, ηp^2^ = 0.13, as well as for PE, *F* (3, 96) = 7.75, *p* < 0.001, ηp^2^ = 0.19; EE, *F* (3, 96) = 7.37, *p* < 0.001, ηp^2^ = 0.18; ATT, *F* (3, 96) = 5.98, *p* < 0.001, ηp^2^ = 0.15; and RLE, *F* (3, 96) = 14.81, *p* < 0.001, ηp^2^ = 0.31. At baseline, no significant between-group differences were detected for AB or any subdimension (all *p* > 0.05), supporting baseline comparability. At both T_1_ and T2, the control group consistently showed higher burnout scores than all intervention groups (all *p* < 0.05). Moreover, the DMG demonstrated the lowest burnout scores among the intervention groups at both T1 and T2, indicating the greatest and most sustained reduction in academic burnout. Although both the DG and MG improved relative to baseline, the combined intervention produced the greatest and most sustained reductions in burnout symptoms.

## Discussion

The present study examined the effects of folk dance, mindful awareness, and their combined application on academic burnout among left-behind children in rural China. The results indicated that all three interventions effectively reduced academic burnout, with the combined intervention (DMG) producing the most significant and sustained improvements. These findings support the theoretical rationale for integrating folk dance and mindful awareness, suggesting that cognitive–emotional regulation and embodied social movement may operate through complementary pathways to enhance psychological resilience and academic engagement. Rather than functioning as isolated strategies, the two approaches appear to converge on shared mechanisms, including emotion regulation, stress buffering, and mind-body integration.

The findings of the present study indicate that folk dance interventions significantly alleviated academic burnout, which is consistent with previous research (Duberg et al., 2020). Folk dance integrates rhythm, coordinated movement, and group interaction, creating opportunities for emotional expression and social connectedness (Margariti et al., 2012). The pleasurable and engaging nature of dance can enhance mood and reduce stress, partly through the release of endorphins associated with rhythmic physical activity (Quiroga Murcia et al., 2010). However, the follow-up results of the present study suggest that the benefits of dance alone may gradually diminish over time. One possible explanation is that dance primarily enhances mood and social engagement through physiological arousal and interpersonal synchrony but may not sufficiently facilitate sustained cognitive restructuring or deeper emotional regulation skills. Although embodied movement can temporarily elevate affect and reduce stress, the absence of structured reflection or skill internalization may limit the consolidation of these gains. Furthermore, once the structured group sessions concluded, students returned to academically demanding environments without continued opportunities for guided practice or reinforcement, which may have contributed to the gradual attenuation of the intervention effects. These findings suggest that while folk dance provides an engaging and emotionally activating experience, its long-term effectiveness may depend on sustained practice opportunities, progressive skill development, and integration into students’ everyday coping strategies. Future dance-based interventions may therefore benefit from incorporating structured progression, periodic reinforcement sessions, and contextual adaptations to students’ developmental and cultural backgrounds.

The results of this study also demonstrate that mindful awareness interventions can effectively reduce academic burnout among left-behind children, which is consistent with previous findings (Zeidan et al., 2010). Mindful awareness primarily cultivates the self-regulation of thoughts and emotions, thereby enhancing attentional control, reducing anxiety, and promoting adaptive coping strategies (Hofmann and Gómez, 2017; Tang et al., 2022). From a neurocognitive perspective, mindfulness practices have been shown to modulate activity in attention-related brain networks and reduce maladaptive responses to negative self-related beliefs (Goldin and Gross, 2010). For children experiencing emotional stress associated with parental separation, such mechanisms may strengthen long-term psychological resilience and decrease vulnerability to academic burnout. Moreover, mindfulness practices may promote self-compassion, which reduces harsh self-judgments that are commonly associated with academic stress and burnout (Neff, 2003). Nevertheless, the relatively smaller improvements observed in the mindful awareness group suggest certain limitations. Mindfulness practices require sustained attentional engagement and a degree of metacognitive awareness, which may be challenging for younger children or those experiencing chronic emotional stress. The abstract and introspective nature of mindfulness exercises may also reduce engagement for students who struggle with concentration or emotional articulation. Without sufficient guidance, repetition, or contextual reinforcement, the internalization of mindfulness skills may remain limited. In addition, the benefits of mindfulness may emerge gradually over time, meaning that relatively short intervention periods may not fully capture its longer-term regulatory effects.

The superior effectiveness of the combined dance-mindful awareness intervention (DMG) suggests the presence of synergistic mechanisms that extend beyond the independent effects of either intervention. Conceptually, this integration reflects a bidirectional enhancement process. Mindful awareness provides structured training in attentional control and emotional regulation, which may stabilize and consolidate the positive affective activation elicited through dance. Conversely, the rhythmic movement, physical activation, and interpersonal synchrony inherent in folk dance create an embodied and socially engaging context that enhances motivation and experiential engagement, thereby facilitating the application of mindful skills in real-time interaction. Rather than functioning as parallel components, the two modalities appear to reinforce one another across cognitive, emotional, and physiological domains. Empirical evidence supports this complementary framework. Dance-based activities have been associated with increased cerebral blood flow and improved white matter integrity (Nilsson et al., 2020; Tait et al., 2017), suggesting potential neurobiological benefits related to cognitive flexibility and emotional processing. In parallel, mindfulness practices have been shown to enhance self-reflection, emotional balance, and metacognitive monitoring (Hölzel et al., 2011). Together, these mechanisms may contribute to both immediate improvements in emotional wellbeing and longer-term regulatory stability, thereby reducing academic burnout more effectively than either intervention alone. Furthermore, the combined intervention appears to address both intrapersonal and interpersonal dimensions of academic burnout. Mindful awareness cultivates internal self-regulation, whereas dance promotes positive affect, rhythmic coordination, and social connectedness. By simultaneously targeting self-regulatory capacity and relational attunement, the integrated model represents a coherent mind–body intervention framework rather than a simple additive approach. Consistent with prior research demonstrating that the integration of contemplative and movement-based practices can enhance engagement and emotional flexibility among school-aged children (Lau and Hue, 2011), the present findings suggest that even a relatively short-term, school-based integrative program can significantly reduce academic burnout among left-behind children—an emotionally vulnerable and socially underserved population.

Beyond its theoretical contributions, this study also has important practical implications. Schools serving left-behind children often lack specialized psychological support resources, making low-cost, group-based interventions particularly valuable. The combined dance-mindful awareness program represents an accessible and culturally adaptable approach that can be integrated into regular school schedules. By simultaneously fostering emotional regulation, social connectedness, and embodied engagement, such integrative programs may provide a feasible strategy for reducing academic burnout and strengthening psychological resilience among vulnerable youth. Policymakers and educators may therefore consider incorporating movement-based and mindfulness-informed activities into school mental health initiatives, particularly in rural or underserved communities where psychosocial support systems are limited.

## Limitations and future direction

Despite its contributions, several limitations should be acknowledged. First, the relatively modest sample drawn from a specific rural region in China may limit the generalizability of the findings. Given the heterogeneity among left-behind children in caregiving arrangements, duration of parental absence, and socioeconomic background, the results may not fully represent other regional, cultural, or educational contexts. Second, academic burnout was assessed primarily through self-report measures. Although the instrument demonstrated satisfactory psychometric properties, self-reported data may be influenced by social desirability bias, response tendencies, and limited emotional literacy, particularly among vulnerable youth. In addition, existing instruments may not fully capture the complex and multidimensional stress experiences of left-behind children, whose academic difficulties are often intertwined with attachment-related challenges and prolonged parental separation. Third, although follow-up assessments were conducted, the duration of follow-up was relatively limited. As academic burnout is shaped by ongoing environmental and developmental pressures, the long-term durability of intervention effects remains to be examined over extended time frames.

Future research should recruit larger and more diverse samples across different regions to enhance external validity. Incorporating multi-method assessment strategies–including teacher reports, behavioral observations, and physiological indicators–would strengthen measurement validity and provide a more comprehensive evaluation of intervention effects. Extending the follow-up period would help clarify long-term developmental trajectories and the sustainability of outcomes. Additionally, examining potential mediators and moderators, such as age, gender, attachment patterns, and caregiving stability, may facilitate a more nuanced understanding of differential responsiveness to intervention components. Such efforts would contribute to the development of more tailored, developmentally sensitive, and contextually appropriate intervention models for vulnerable populations.

## Conclusion

Overall, the findings indicate that the combined intervention was more effective in reducing academic burnout than either dance or mindful awareness alone. The results suggest that folk dance produced greater short-term benefits than mindful awareness, though its effects were less sustained, whereas mindful awareness offered smaller but more stable improvements. The combination integrated these advantages, yielding both immediate and enduring reductions in burnout symptoms. These findings underscore the value of multi-modal interventions in supporting the academic and emotional wellbeing of left-behind children.

## Data Availability

The raw data supporting the conclusions of this article will be made available by the authors, without undue reservation.
