# Supplementary material for: Effects of mindful awareness intervention folk-dance intervention and combined intervention on academic burnout in left-behind children in rural China
Source: Front Psychol. 2026 Apr 27;17:1745058. doi: 10.3389/fpsyg.2026.1745058 (PMC13159487; doi:10.3389/fpsyg.2026.1745058)
Supplement: Supplementary file 1 [file Data_Sheet_1.pdf]

Variable	Item	Factor loading	AVE	CR	Cronbach's α
Physical Exhaustion	PE1	0.657	0.504	0.802	0.809
	PE2	0.716			
	PE3	0.726			
	PE4	0.715			
Emotional Exhaustion	EE1	0.683	0.496	0.886	0.871
	EE2	0.63			
	EE3	0.643			
	EE4	0.739			
	EE5	0.781			
	EE6	0.738			
	EE7	0.656			
	EE8	0.751			
Attitudes toward Learning	ATT1	0.667	0.508	0.805	0.878
	ATT2	0.757			
	ATT3	0.715			
	ATT4	0.710			
Reduced Learning Efficacy	RLE1	0.696	0.448	0.800	0.820
	RLE2	0.539			
	RLE3	0.629			
	RLE4	0.732			
	RLE5	0.732			

Supplementary Table 1 Results of AVE and CR ofthe Model of the Learning Burnout Scale

The convergent validity and Cronbach's α of the questionnaire are presented in Supplementary Table1. All items across dimensions demonstrate factor loadings>0.6, Cronbach's α>0.7, and Average Validity Excess (AVE)>0.4, indicating that the Learning Burnout Scale possesses acceptable convergent validity and reliability.

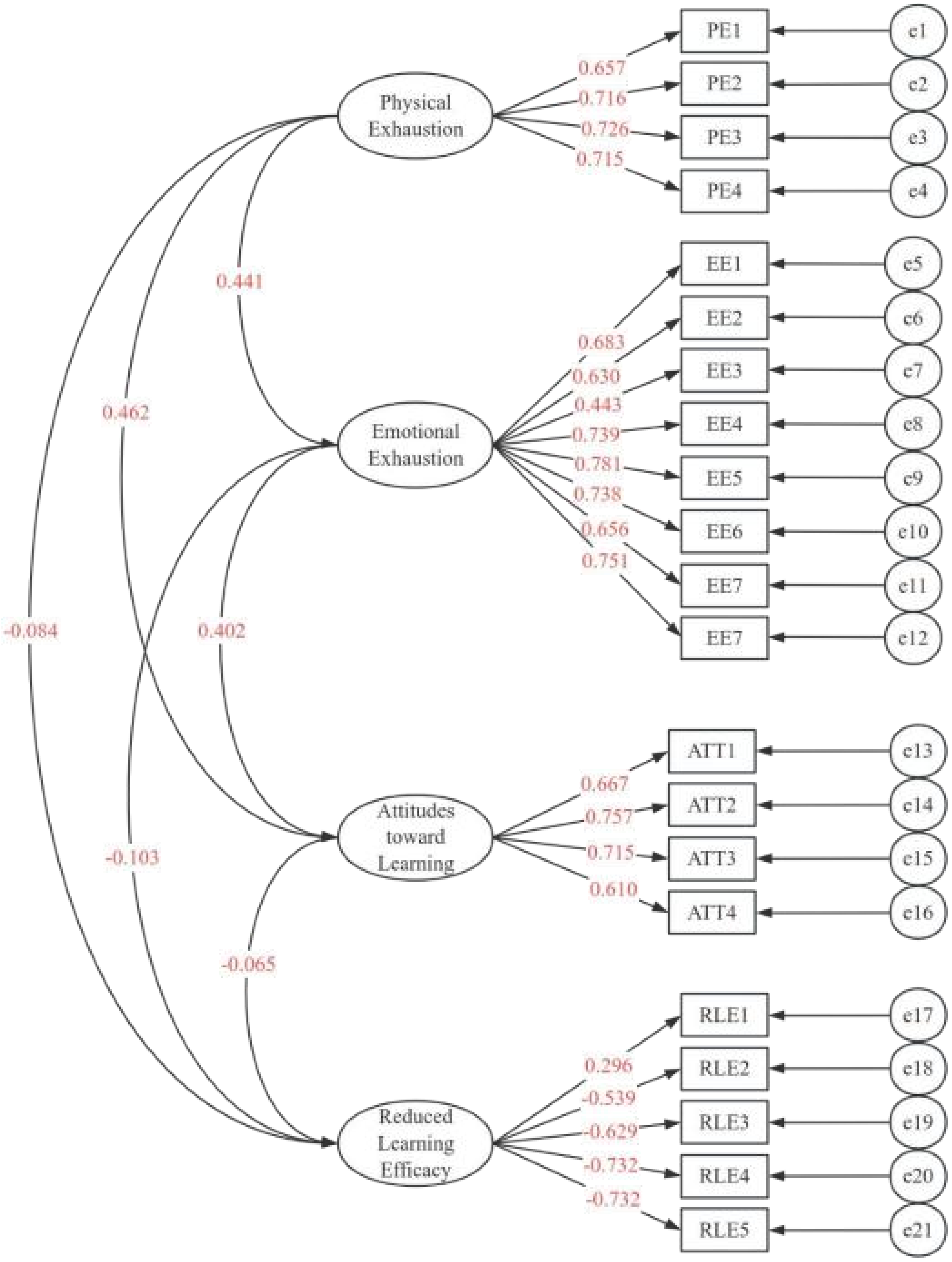

Supplementary Figure 1 Model diagram of confirmatory factor analysis for the Learning Burnout Scale

The model demonstrated excellent fit to the data ($\chi 2$ / df = 4.044, RMSEA = 0.04, NFI = 0.945 > 0.9, IFI = 0.958 > 0.9, TLI = 0.949 > 0.9, CFI = 0.958 > 0.9,P<0.001), as shown in Supplementary Figure 1

Supplementary Table 2 Discriminant validity of the Learning Burnout Scale

	PE	EE	ATT	RLE
PE	0.709			
EE	0.441**	0.704		
ATT	0.462**	0.402**	0.712	
RLE	0.403**	0.484**	0.465**	0.669

Note: * indicates P<0.05; ** indicates P<0.01, and the underlined values represent the square root of AVE.

The discriminant validity is demonstrated in Supplementary Table 2, where the root of the Average Validity Excess (AVE) consistently exceeds the standardized correlation coefficients outside the diagonal, confirming the Learning Burnout Scale's strong discriminant validity.
